# Impaired lymph node stromal cell function during the earliest phases of rheumatoid arthritis

**DOI:** 10.1186/s13075-018-1529-8

**Published:** 2018-02-26

**Authors:** Janine S. Hähnlein, Reza Nadafi, Tineke de Jong, Tamara H. Ramwadhdoebe, Johanna F. Semmelink, Karen I. Maijer, IJsbrand A. Zijlstra, Mario Maas, Danielle M. Gerlag, Teunis B. H. Geijtenbeek, Paul P. Tak, Reina E. Mebius, Lisa G. M. van Baarsen

**Affiliations:** 1 Amsterdam Rheumatology & immunology Centre (ARC), Department of Clinical Immunology and Rheumatology, Academic Medical Centre, University of Amsterdam, Meibergdreef 9, Amsterdam, 1105 AZ the Netherlands; 2 Department of Experimental Immunology, Academic Medical Centre, University of Amsterdam, Meibergdreef 9, Amsterdam, 1105 AZ the Netherlands; 3 Department of Molecular Cell Biology and Immunology, VU Medical Centre, Amsterdam, the Netherlands; 4 Department of Radiology, Academic Medical Centre, University of Amsterdam, Amsterdam, the Netherlands; 5 Present address: Clinical Unit Cambridge, GlaxoSmithKline, Cambridge, UK; 6 Present address: Ghent University, Ghent, Belgium; 7 Present address: University of Cambridge, Cambridge, UK; 8 Present address: GlaxoSmithKline, Stevenage, UK

**Keywords:** Lymph node stromal cells, Early rheumatoid arthritis, Autoimmunity, Tolerance, Immunity

## Abstract

**Background:**

Systemic autoimmunity can be present years before clinical onset of rheumatoid arthritis (RA). Adaptive immunity is initiated in lymphoid tissue where lymph node stromal cells (LNSCs) regulate immune responses through their intimate connection with leucocytes. We postulate that malfunctioning of LNSCs creates a microenvironment in which normal immune responses are not properly controlled, possibly leading to autoimmune disease. In this study we established an experimental model for studying the functional capacities of human LNSCs during RA development.

**Methods:**

Twenty-four patients with RA, 23 individuals positive for autoantibodies but without clinical disease (RA risk group) and 14 seronegative healthy control subjects underwent ultrasound-guided inguinal lymph node (LN) biopsy. Human LNSCs were isolated and expanded in vitro for functional analyses. In analogous co-cultures consisting of LNSCs and peripheral blood mononuclear cells, αCD3/αCD28-induced T-cell proliferation was measured using carboxyfluorescein diacetate succinimidyl ester dilution.

**Results:**

Fibroblast-like cells expanded from the LN biopsy comprised of fibroblastic reticular cells (gp38^+^CD31^−^) and double-negative (gp38^−^CD31^−^) cells. Cultured LNSCs stably expressed characteristic adhesion molecules and cytokines. Basal expression of C-X-C motif chemokine ligand 12 (CXCL12) was lower in LNSCs from RA risk individuals than in those from healthy control subjects. Key LN chemokines C-C motif chemokine ligand (CCL19), CCL21 and CXCL13 were induced in LNSCs upon stimulation with tumour necrosis factor-α and lymphotoxin α_1_β_2_, but to a lesser extent in LNSCs from patients with RA. The effect of human LNSCs on T-cell proliferation was ratio-dependent and altered in RA LNSCs.

**Conclusions:**

Overall, we developed an experimental model to facilitate research on the role of LNSCs during the earliest phases of RA. Using this innovative model, we show, for the first time to our knowledge, that the LN stromal environment is changed during the earliest phases of RA, probably contributing to deregulated immune responses early in disease pathogenesis.

**Electronic supplementary material:**

The online version of this article (10.1186/s13075-018-1529-8) contains supplementary material, which is available to authorized users.

## Background

The earliest stages of rheumatoid arthritis (RA) are characterized by the presence of RA-specific autoantibodies such as rheumatoid factor (RF) and anti-citrullinated protein antibodies (ACPAs) years before the manifestation of clinical disease [1]. In the Amsterdam health care region, ACPA-positive individuals with arthralgia have an approximately 50% chance of developing RA within 3–4 years [2, 3]. Interestingly, during this at-risk phase synovial inflammation as determined by immunohistochemistry seems absent, suggesting that infiltration of the synovial tissue by inflammatory cells occurs in a later stage [4, 5]. Because systemic autoimmunity seems to precede synovial tissue inflammation, other, as yet unidentified immune processes, possibly outside synovial tissues, are altered and contribute to disease development.

To effectively mount an adaptive immune response, secondary lymphoid tissues are essential. Animal models have shown phenotypic changes in the cellular compartment of peripheral lymph nodes (LNs) before the onset of arthritis [6]. Recently, we detected altered frequencies of B cells, T-cell subsets and innate lymphoid cell subsets in LN biopsies of subjects with RA risk and patients with early-stage RA when compared with healthy control subjects [7–10], indicating that LN activation was already present during the RA risk phase. Studies in mouse models revealed that lymph node stromal cells (LNSCs) play an important role in the regulation of T- and B-cell responses [11, 12]. LNSCs physically construct the LN, and through production of chemokines and adhesion molecules, they guide immune cells within the LN [13–15]. In addition, LNSCs produce cytokines important for lymphocyte activation, differentiation and survival [16]. In mouse models, LNSCs have been found to induce peripheral T-cell tolerance by direct antigen presentation and clonal deletion as well as maintenance of regulatory T cells [17–19]. Furthermore, during immune responses they are capable of suppressing T-cell proliferation independently of antigens [19–21]. Accordingly, LNSCs are key players in immunity and tolerance. We hypothesise that malfunctioning of LNSCs leads to a microenvironment where immune responses are not properly controlled, which may lead to the activation of (autoreactive) lymphocytes and production of autoantibodies. LNSCs have been studied mainly in animal models, because so far human LNSCs have been obtained either from whole LNs removed during surgery or from deceased individuals [22–24]. Isolating and sorting sufficient numbers of LNSCs directly ex vivo is technically challenging [24]. We therefore aimed to develop an experimental model to allow research on human LNSCs during health and RA and to lay the foundation for further research on these immune-shaping cells.

## Methods

### Study subjects and lymph node biopsy sampling

Individuals with arthralgia and elevated immunoglobulin M (IgM)-RF and/or ACPA levels but without any evidence of arthritis upon examination were included (individuals with RA risk phase C/D) [25]. Median follow-up of individuals with RA risk was 20.3 months (IQR 12.9–33.2), and none of the individuals with RA risk developed arthritis during this period. In addition, patients with RA with established disease based on fulfilment of the American College of Rheumatology/European League Against Rheumatism 2010 criteria [26] and as assessed by the rheumatologist, as well as healthy control subjects without any joint complaints and without elevated IgM-RF and/or ACPA levels, were included. To be eligible, the healthy control subjects could not have an active viral infection or any history of autoimmunity or malignancy and no present or previous use of disease-modifying anti-rheumatic drugs, biologics or other experimental drugs. IgM-RF was measured using an IgM-RF enzyme-linked immunosorbent assay (ELISA) (upper limit of normal [ULN] 49 kU/L [kilo Unit/L]; HYCOR Biomedical, Garden Grove, CA, USA). ACPA were measured using the CCPlus anti-cyclic citrullinated peptide 2 ELISA (ULN 25 kAU/L [kilo arbitrary Unit/L]; Euro Diagnostica, Malmö, Sweden). The study was performed according to the principles of the Declaration of Helsinki and approved by the institutional medical ethical review board of the Academic Medical Centre, and all study subjects gave written informed consent. All study subjects underwent an ultrasound-guided inguinal LN needle core biopsy as previously described [27]. Table 1 shows the demographics of the included subjects.

**Table 1:** Demographic data of study subjects

	Healthy control subjects (*n* = 14)	Individuals with RA risk (*n* = 23)	Patients with RA (*n* = 24)
Female sex, n (%)	9 (64)	20 (87)	17 (70)
Median age, years, (IQR)	29 (26–37)	49 (35–57)	56 (44–61)
IgM-RF-positive, n (%)	0 (0)	10 (43)	20 (3–107)
IgM-RF level, kU/L, median (IQR)	–	20 (3–107)	131 (31–309)
ACPA-positive, n (%)	0 (0)	13 (57)	18 (75)
Median ACPA level, kAU/L (IQR)	–	43 (4–177)	115 (21–924)
IgM-RF and ACPA both positive, n (%)	0 (0)	0 (0)	14 (58)
Median DAS28, (IQR)	–	–	5 (1–10)^c^
Median ESR, mm/h (IQR)	–	7 (2–10)	11 (5–27)^b^
Median CRP, mg/L (IQR)	0.5 (0.3–1.2)^c^	1.6 (0.9–3.2)	4.6 (1.4–13)^d^
Median TJC68 (IQR)	0 (0)	1.5 (0–4.5)	9 (4–20)^e^
Median SJC68 (IQR)	0 (0)	0 (0)	5 (1–10)^d^
Treatment, n (%)			9 (39)
Corticosteroids			6 (26)
NSAIDs			4 (17)^f^
DMARDs			5 (22)
Failed TNF inhibitor therapy			5 (22)

*Abbreviations: RA Rheumatoid arthritis, IgM-RF* immunoglobulin M rheumatoid factor, *kU/L* kilo Unit/L, *ACPA* anti-citrullinated protein antibodies, *kAU/L* kilo arbitrary Unit, *ESR* erythrocyte sedimentation rate, *CRP* C-reactive protein, *TJC68* 68-joint tender joint count, *SJC68* 68-joint swollen joint count, *NSAID* non-steroidal anti-inflammatory drug, *DMARD* disease-modifying antirheumatic drug, *DAS28* disease activity score in 28 joints, *TNF* tumour necrosis factor

^a^ Levels missing from one individual

^b^ Levels missing from two individuals

^c^ Levels missing from six individuals

^d^ Levels missing from seven individuals

^e^ Levels missing from five individuals

^f^ Treatment unknown for five individuals

### Lymph node stromal cell culture

After depletion of lymphocytes through a 70-μm cell strainer (Corning, Landsmeer, the Nederlands), the remaining stromal tissue of a freshly collected LN needle core biopsy was plated on a 6-well culture dish (CELLSTAR®; Greiner Bio-One/VWR, Alpen a/d Rijn, the Nederlands) (passage 0; P0). Complete cell culture medium was added. It consisted of DMEM, low glucose (Thermo Fisher Scientific, Landsmeer, the Netherlands) supplemented with 0.1% penicillin (Astellas Pharma Inc., Leiden, the Netherlands), 0.1% streptomycin, 0.05 mg/ml gentamicin, 10 mM 4-(2-hydroxyethyl)-1-piperazineethanesulfonic acid (HEPES) buffer, and 2 mM l-glutamine (all from Thermo Fisher Scientific), as well as 10% FCS (GE Healthcare, Zeist, the Netherlands). Upon reaching confluence of > 80% cells, were passaged to a T75 tissue culture flask (P1) or into two T225 flasks (P2; both Corning® Costar®; Corning). Before being harvested, cells were washed with sterile warm PBS (Fresenius Kabi, 's-Hertogenbosch, the Netherlands) and incubated with 0.05% trypsin/5 mM ethylenediaminetetraacetic acid (Thermo Fisher Scientific) in PBS for 7 min at 37 °C. Subsequently, an equal amount of complete medium was added, after which the cell suspension was collected and centrifuged for 10 min at 1000 rpm at 4 °C. Cells were resuspended in cold complete medium and counted using trypan blue (Sigma-Aldrich, Zwijndrecht, the Nederlands) in a BRAND® Bürker Türk chamber (Sigma-Aldrich). Human LNSCs (passages 4 to 8) were seeded in a 24-well plate (30,000 cells/well) and stimulated with tumour necrosis factor-α (TNF-α) (5 ng/ml; Life Technologies, Landsmeer, the Nederlands) plus lymphotoxin α_1_β_2_ (50 ng/ml; R&D Systems, Abingdon, UK).

### Flow cytometric analysis

Human LNSCs (passages 3 to 6) were cultured in a 6-well culture dish (100,000 cells/well). To harvest adherent cells, 1 ml of TrypLE™ Select reagent (Thermo Fisher Scientific) was added for 10 min at 37 °C. Subsequently, cells were washed in protein blocking agent (PBA) buffer (PBS containing 0.01% NaN_3_ and 0.5% bovine serum albumin [Sigma-Aldrich]) and stained for 30 min at room temperature protected from light using the following directly labelled antibodies: CD45 fluorescein isothiocyanate (FITC) (clone HI30; BD Diagnostics, Vianen, the Netherlands), podoplanin Alexa Fluor 647 (clone NC-08; BioLegend, London, UK), CD31 allophycocyanin (APC)-eFluor 780 (clone WM-59; eBioscience, Vienna, Austria), human leucocyte antigen A, B, C phycoerythrin-cyanine 7 (PE-Cy7, clone G46–2.6; BioLegend), or corresponding isotype controls. To examine the expression of podoplanin on LNSCs cultured over different passages, cells were stained for 1 h on ice with unconjugated anti-human podoplanin (clone NZ-1; AngioBio, Huissen, the Nederlands), washed, and subsequently incubated with polyclonal goat anti-rat IgG Alexa Fluor 647 (Thermo Fisher Scientific). Cells were washed in PBA and measured using a FACSCanto II flow cytometer (BD Biosciences, Vianen, the Nederlands). Data were analysed using FlowJo software (FlowJo, Ashland, OR, USA).

### Co-cultures containing LNSCs and PBMCs and T-cell proliferation assay

LNSCs (passages 4 to 8) in amounts of 25,000, 10,000, 5000 or 1250 were seeded in duplicates in a 96-well flat-bottomed plate and allowed to rest overnight in DMEM complete culture medium. Subsequently, LNSCs were pre-treated with 50 ng/ml interferon-γ (IFN-γ) (eBioscience) for 48 h or refreshed with DMEM complete medium. Peripheral blood mononuclear cells (PBMCs) that had previously been isolated from healthy donors by using standard density gradient centrifugation and subsequently cryopreserved, were thawed and allowed to rest overnight at 37 °C in RPMI 1640 medium supplemented with 10% FCS (GE Healthcare), 0.1% penicillin (Astellas Pharma), 0.1% streptomycin, 10 mM HEPES buffer and 2 mM l-glutamine (all from Life Technologies). Then, PBMCs were washed and labelled with 2 μl of carboxyfluorescein diacetate succinimidyl ester (CFDA-SE) FITC (clone C1157; Life Technologies) in PBS for 8 min at 37 °C. After removing DMEM complete medium and washing LNSCs once with warm PBS, 50,000 labelled PBMCs in RPMI complete medium per 96-well chamber were added to LNSCs, resulting in ratios of 1:2, 1:5, 1:10 and 1:40 LNSCs to PBMCs. Simultaneously, PBMCs were stimulated with anti-CD3 (1:10,000, clone 1XE; Sanquin, Amsterdam, the Netherlands) and anti-CD28 (0.25 μg/ml, clone 15E8; Sanquin). Cultures were harvested 96 h later, washed with PBA buffer and stained for 30 min at room temperature protected from light using the following directly labelled antibodies: CD45 V500 (clone HI30; BD Biosciences), CD4 PE-Cy7 (clone SK3; eBioscience) and CD8a APC-eFluor 780 (clone SK1; eBioscience). Cells were washed in PBA and measured using the FACSCanto II flow cytometer. Data were analysed using FlowJo software. This methodology was set up by testing PBMCs isolated from four different healthy donors, whereas for the subsequent co-culture experiments, PBMCs from one healthy donor were selected to enable direct comparison between LNSCs from different donors.

### qRT-PCR

Messenger RNA (mRNA) was isolated using the RNeasy Mini Kit or the RNeasy Micro Kit (Qiagen, Venlo, the Netherlands) according to the manufacturer’s instructions, including a DNase step to remove genomic DNA. Subsequently complementary DNA (cDNA) was prepared using the RevertAid H Minus First Strand cDNA Synthesis Kit (Thermo Fisher Scientific). qRT-PCR was performed using either TaqMan® Universal PCR Master Mix combined with TaqMan assays or SYBR® Green PCR Master Mix (all from Thermo Fisher Scientific) combined with primers made in-house (Thermo Fisher Scientific). The TaqMan assays and primer sequences are described in Additional file 1: Table S1. For detection, we used the StepOnePlus™ Real-Time PCR System (Thermo Fisher Scientific). Values for each gene were normalized to the expression level of 18S ribosomal RNA. An arbitrary calibrator sample was used for normalization. For calculating the relative quantity, the 2^−ΔΔ*Ct*^ comparative cycle threshold method was used for TaqMan assays, and a standard curve method was applied for SYBR Green assays.

### Nitric oxide measurement

Nitric oxide (NO) was measured by evaluating the nitrite content in culture media using modified Griess reagent (G4410; Sigma-Aldrich) according to the manufacturer’s instructions. The co-culture supernatant (100 μl) of healthy individuals with RA risk and of patients with RA was mixed with the same volume of Griess reagent for 5 min, and absorbance was measured at 540 nm. A standard curve with increasing concentrations of sodium nitrite (NaNO_2_) was constructed in parallel and used for quantitation.

### Statistics

Data are presented as median with IQR or mean with SD when normally distributed. Differences between study groups were analysed using the Kruskal-Wallis test followed by Dunn’s post hoc test or two-way analysis of variance followed by Dunnett’s multiple comparisons test, where appropriate. Prism software version 7.01 (GraphPad Software, La Jolla, CA, USA) was used for statistical analysis. *P* values < 0.05 were considered statistically significant.

## Results

### Phenotyping of human LNSCs

**Fig. 1:**
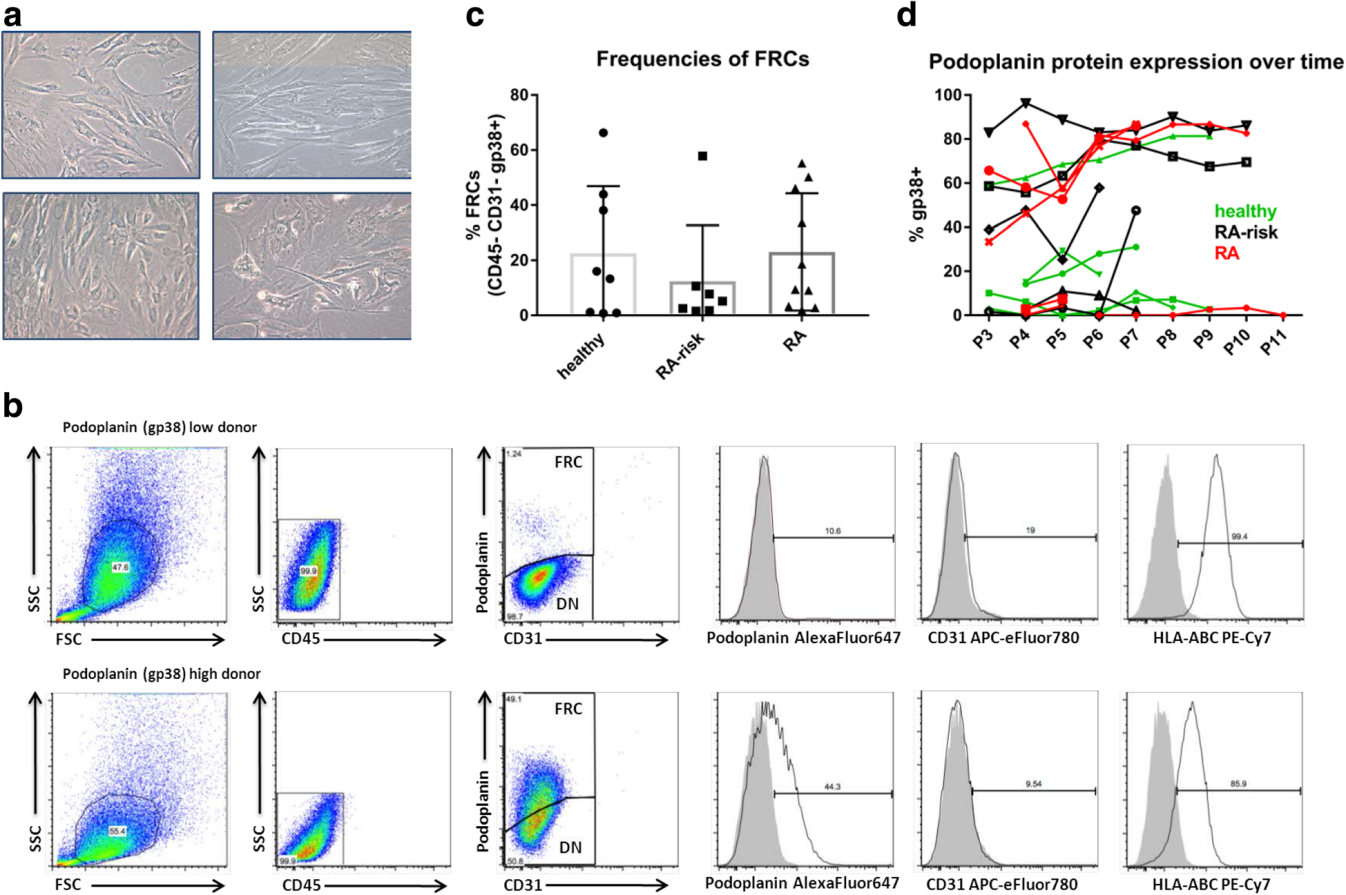
Morphologic and phenotypic characterization of cultured human lymph node stromal cells (LNSCs). **a** Cells growing out of the biopsy were mainly fibroblastic and formed dense networks. During growth, human LNSCs also started to branch and stretch or showed a more roundish morphology. **b** Flow cytometric analysis based on the expression of CD45, podoplanin (gp38) and CD31. Cells in culture (passages 3 to 6 [P3–P6]) were double-negative (DN) cells and fibroblastic reticular cell (FRCs). Gating was based on isotype controls. Human leucocyte antigen A, B, C (HLA-ABC) expression served as a positive control. Representative figures of 2 donors out of 25 experiments are shown. **c** Frequencies of FRCs (CD45^−^CD31^−^ podoplanin [gp38]^+^) measured by fluorescence-activated cell sorting as described in (**b**) (P3–P6; *n* = 25) in different donor groups. **d** Follow-up of podoplanin (gp38) expression over different culture passages as measured by flow cytometry in a different cohort of 16 donors (healthy, *n* = 5; rheumatoid arthritis [RA] risk, *n* = 5; and RA, *n* = 6). APC Allophycocyanin, Cy Cyanine, FSC Forward scatter, PE Phycoerythrin, SSC Side scatter

Owing to the small size of the obtained LN needle biopsies, digestion and immediate sorting of LNSCs did not yield sufficient numbers of LNSCs for direct analyses. However, LNSCs were capable of growing in vitro from freshly obtained inguinal LN biopsies. Mainly fibroblast-like cells expanded easily, and in some donors LNSCs showed network formation (Fig. 1a). Expanded LNSCs often contained a mixed morphology consisting of fibroblastic as well as more roundish cells, which was unrelated to disease state. Whereas most LNSCs needed approximately 1–3 weeks to reach confluence between passages, LNSCs from a few donors required longer or failed to grow (11 of 136 donors). Overall, this culture system enabled a relatively easy, although slow, expansion of human LNSCs.

Expanded human LNSCs in culture (passages 3–6) consisted primarily of a mixture of double-negative cells (DN; CD45^−^podoplanin^−^CD31^−^) and fibroblastic reticular cells (FRCs; CD45^−^podoplanin^+^CD31^−^) as reported previously [24] (Fig. 1b and c). The variation of podoplanin-expressing cells within cultured human LNSCs was similar between donor groups (Fig. 1c). Podoplanin expression showed some variation in and between donors over consecutive passages, but without a consistent trend towards loss or increase across passages (Fig. 1d), which corresponds to continuous podoplanin expression reported previously in human lymphatic endothelial cells [28]. Furthermore, the frequency of podoplanin-positive cells did not correlate with any clinical parameters, such as autoantibodies or age (data not shown).

### LNSCs of patients with RA are less capable of inducing key LN chemokines CCL19 and CXCL13

**Fig. 2:**
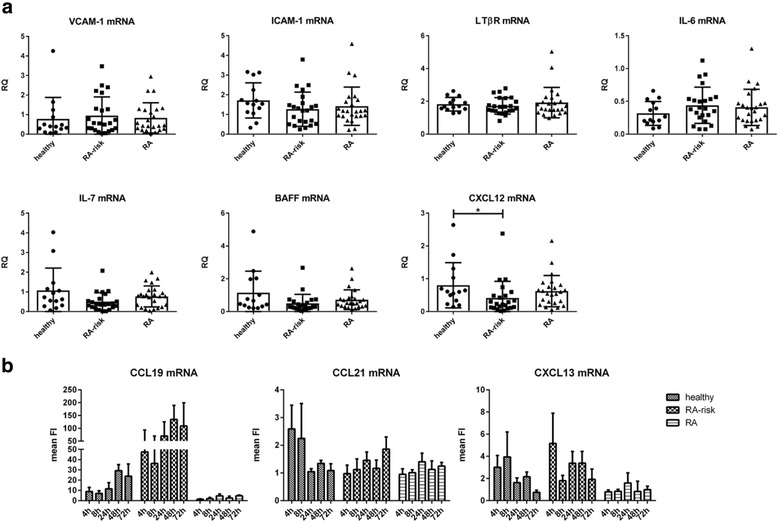
Expression of genes characteristic for lymph node stromal cells (LNSCs). **a** Expression of genes in LNSCs of passage 2 was assessed by qPCR and compared between different donor groups (healthy, *n* = 14; individuals with rheumatoid arthritis [RA] risk, *n* = 23; patients with RA, *n* = 24). Relative quantity (RQ) is displayed as median and IQR. Differences between donor groups were assessed by the Kruskal-Wallis test followed by Dunn’s post hoc test. * *P* < 0.050. **b** Expression of lymphoid chemokines was assessed after stimulation with tumour necrosis factor-α (TNFα)  and lymphotoxin α_1_β_2_ for 4 h, 8 h, 24 h, 48 h and 72 h by qPCR in LNSCs (passages 4 to 8). Mean fold induction (FI) and SD of *n* = 5 per donor group are shown. Donor characteristics are listed in Table 2. *BAFF* B-cell-activating factor, CCL C-C motif chemokine ligand, CXCL C-X-C motif chemokine ligand, ICAM-1 Intercellular adhesion molecule 1, IL Interleukin, *mRNA* Messenger RNA, VCAM-1 Vascular cell adhesion molecule 1

The expression of characteristic LNSC-related genes such as vascular cell adhesion molecule 1 (VCAM-1), intercellular adhesion molecule 1 (ICAM-1), lymphotoxin-β receptor (LTβR), interleukin (IL)-7, B-cell-activating factor (BAFF) and IL-6 in LNSCs (all from passage 2) was highly variable but showed no significant differences between donor groups and also not when stratified for ACPA status. Only C-X-C motif chemokine ligand 12 (CXCL12) showed a significantly lower expression in LNSCs from individuals with RA risk compared with healthy control subjects (*P* = 0.0155) (Fig. 2a). To investigate whether these LNSC characteristics changed during culturing, we cultured LNSCs over several passages. We detected no significant changes in mRNA levels of IL-7, VCAM-1, ICAM-1 and podoplanin between passages 0 and 12 (*n* = 18) (Additional file 1: Figure S1). Furthermore, we found no correlation between the expression of genes analysed under homeostatic conditions in P2 LNSCs with clinical parameters such as age or autoantibody titres. When correlated with podoplanin mRNA levels at P2 measured in total LNSC cultures, we detected solely a significant, although weak, correlation with IL-7 mRNA (*P* ≤ 0.0001; Spearman’s *r* = 0.534) (Additional file 1: Figure S2).

As anticipated, mRNA levels of C-C motif chemokine ligand 19 (CCL19), CCL21 and CXCL13 in LNSCs were low or undetected in LNSCs under homeostatic conditions, but stimulation with TNF-α plus lymphotoxin α_1_β_2_ rapidly induced these key LN chemokines. TNF-α and lymphotoxin α_1_β_2_, produced by lymphocytes, are key factors in the cross-talk between LNSCs and lymphocytes and in regulation of LN organogenesis, homeostasis and remodelling, and they are known to induce the expression of these critical characteristic chemokines produced by LNSCs [29–31] (Fig. 2b) (*n* = 5 per donor group; donor characteristics listed in Table 2).

**Table 2:** Demographic data of study subjects used in tumour necrosis factor-α and lymphotoxin α_1_β_2_ stimulation experiments

	Healthy control subjects (*n* = 5)	Individuals with RA risk (*n* = 5)	Patients with early RA^a^ (*n* = 5)
Passages	P4–P6	P5–P8	P6–P7
Female sex, n (%)	5 (100)	5 (100)	4 (80)
Median age, years (IQR)	28 (26–40)	51 (49–57)	35 (28–58)
IgM-RF-positive, n (%)	0 (0)	4 (80)	3 (60)
IgM-RF level, kU/L, median (IQR)	–	3 (1–42)	6 (3–288)
ACPA-positive, n (%)	0 (0)	4 (80)	4 (80)
ACPA level, kAU/L, median (IQR)	–	47 (16–343)	54 (22–1563)
IgM-RF and ACPA both positive, n (%)	0 (0)	0 (0)	2 (40)
DAS28, median (IQR)	–	–	3.9 (3–7)
CRP, mg/L, median (IQR)	0.4 (0.3–2.7)	3.2 (1.9–4.4)	3 (0.9–100.4)

*Abbreviations*: *ACPA* anti-citrullinated protein antibodies, *kAU/L* kilo arbitrary Unit/L, *CRP* C-reactive protein, *DAS28* disease activity score in 28 joints, *IgM-RF* immunoglobulin M-rheumatoid factor, *kU/L* kilo Unit/L, *RA* rheumatoid arthritis

^a^ Patients with early RA: naïve for disease-modifying anti-rheumatic drugs and biologics with a disease duration (defined by arthritis in any joint) less than 1 year

Of interest, CCL19 and CXCL13 levels were significantly differentially induced between the donor groups (*P* = 0.0018 for CCL19 and *P* = 0.0155 for CXCL13), with lower induction observed in LNSCs obtained from patients with RA. This stimulation also strongly induced the expression of VCAM-1 and ICAM-1, and to a lesser extent IL-7 and podoplanin (Additional file 1: Figure S3). We found no correlation between the induction of these chemokines after stimulation with clinical parameters such as age or autoantibody titres. Moreover, all observed inductions of chemokines and characteristic genes were independent of basal podoplanin protein expression measured by fluorescence-activated cell sorting, indicating that human DN cells and FRCs share these common characteristics. However, the induction of ICAM-1 mRNA correlated strongly with the induction of podoplanin mRNA (Additional file 1: Figure S4).

### The effect of human LNSCs on T-cell proliferation is ratio-dependent and altered in RA LNSCs

**Fig. 3:**
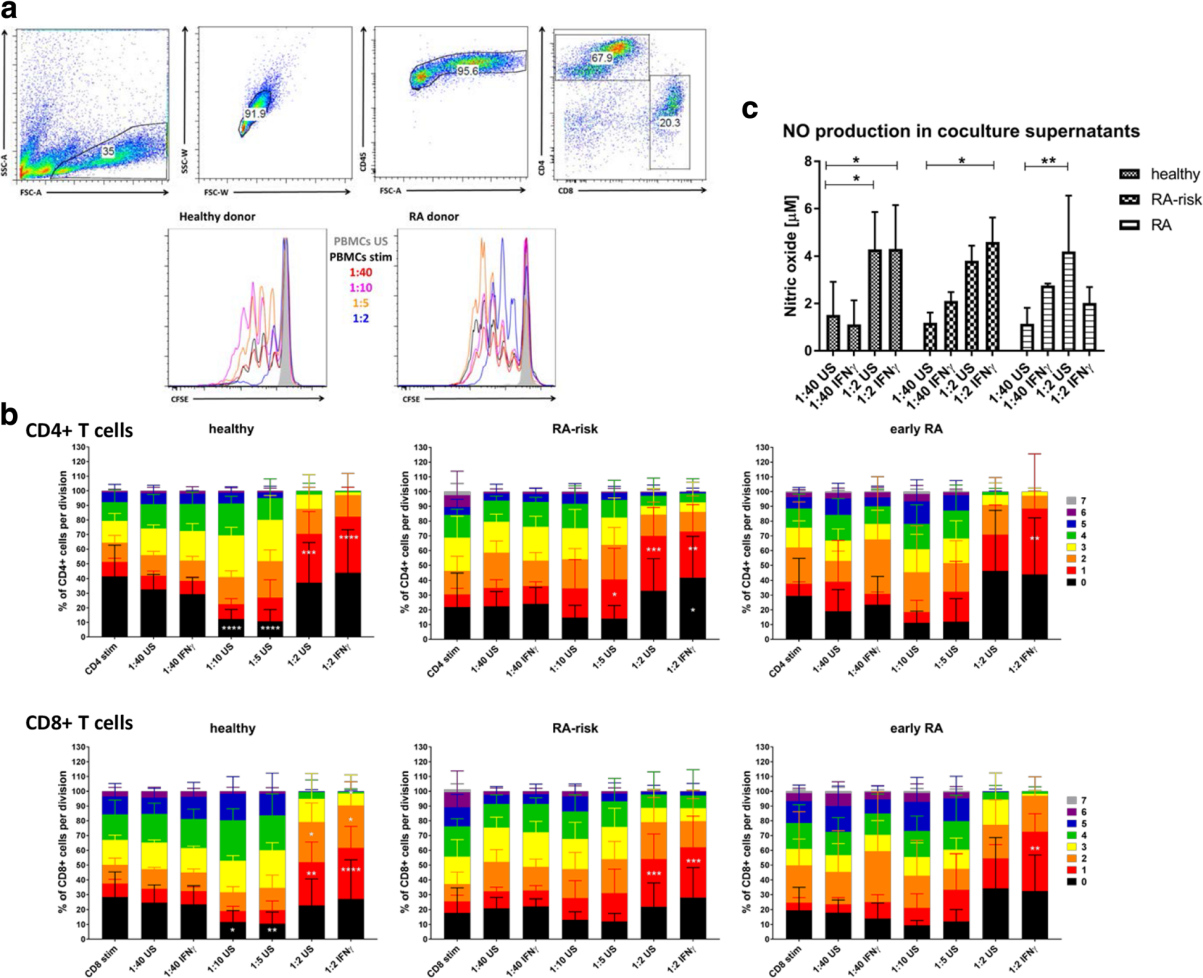
Regulation of T-cell proliferation by lymph node stromal cells (LNSCs). **a** Flow cytometry gating strategy used to identify CD4^+^ and CD8^+^ T-cell subsets according to CD45, CD4 and CD8 expression. Numbers adjacent to the outlined areas indicate percentages of cells in the gated population. A representative carboxyfluorescein succinimidyl ester (CSFE) dilution plot is shown for 2 of 15 donors. **b** Proliferation of CSFE-labelled CD4^+^ and CD8^+^ T cells out of 50,000 peripheral blood mononuclear cell (PBMCs) (all from one donor) activated with αCD3 and αCD28 for 96 h without LNSCs (passages 4 to 8) or co-cultured with 1250 (1:40), 5000 (1:10), 10,000 (1:5) or 25,000 LNSCs (1:2). LNSCs were cultured from healthy donors, individuals with RA risk and patients with RA and pre-treated with interferon-γ (IFN-γ) or not. Data are presented as the percentage of total cells found in the respective cell division (mean and SD of *n* = 5 donors per group; donor characteristics listed in Table 3). Upper panels show CD4^+^ T-cell data; lower panels show data of CD8^+^ T cells displayed co-cultured together with LNSCs of respective donor groups. Differences between T cell with or without LNSCs were assessed using two-way analysis of variance ANOVA. * *P* < 0.050, ** *P* < 0.010, *** *P* < 0.001, **** *P* < 0.0001 **c** Nitric oxide (NO) production in co-culture supernatants was measured after 96 h of co-culture using Griess reagent. Mean and SD of 10 donors (healthy, *n* = 5; individuals with RA risk, *n* = 3; patients with RA, *n* = 2) are shown. Differences between conditions were assessed using two-way ANOVA. * *P* < 0.050, ** *P* < 0.010. FSC-A Forward scatter area, FSC-W Forward scatter width, SSC-A Side scatter area, SSC-W Side scatter width

Finally, we aimed to investigate the effect of human LNSCs on T-cell proliferation. Therefore, we performed a co-culture experiment using a fixed number of 50,000 activated (anti-CD3 and anti-CD28) allogeneic PBMCs derived from one healthy donor together with increasing numbers of LNSCs (passages 4 to 8) from the different study groups (Fig. 3). This way we were able to compare the impact of LNSCs on T-cell proliferation between healthy donors, individuals with RA risk and patients with RA. Donors (*n* = 5 per study group; donor characteristics listed in Table 3) were age- and sex-matched. All donors with RA risk were ACPA^+^/RF^−^, whereas patients with RA were ACPA^+^RF^−^ or double-positive.

**Table 3:** Demographic data of study subjects used in co-culture experiments

	Healthy control subjects (*n* = 5)	Individuals with RA risk (*n* = 5)	Patients with early RA^a^ (*n* = 5)
Passages	P4–P8	P4–P8	P4–P8
Female sex, n (%)	3 (60)	3 (60)	4 (80)
Median age, years (IQR)	28 (24–33)	50 (28–57)	47 (35–57)
IgM-RF-positive, n (%)	0 (0)	0 (0)	4 (80)
IgM-RF level, kU/L, median (IQR)	–	3 (1–15.5)	437 (159–2210)
ACPA-positive, n (%)	0 (0)	5 (100)	5 (100)
ACPA level, kAU/L, median (IQR)	–	75 (45–112.5)	328 (61–1969)
IgM-RF and ACPA both positive, n (%)	0 (0)	0 (0)	4 (80)
DAS28, median (IQR)	–	–	5.3 (3.2–6.8)
CRP, mg/L, median (IQR)	0.4 (0.3–2.7)	1.6 (0.7–2)	4.4 (1.3–19.8)

*Abbreviations*: *ACPA* anti-citrullinated protein antibodies,* kAU/L* kilo arbitrary Unit/L, *CRP* C-reactive protein, *DAS28* disease activity score in 28 joints, *IgM-RF* immunoglobulin M-rheumatoid factor,* kU/L* kilo Unit/L, *RA* rheumatoid arthritis

^a^ Patients with early RA: naïve for disease-modifying anti-rheumatic drugs and biologics with a disease duration (defined by arthritis in any joint) less than 1 year

LNSCs in different ratios with unstimulated PBMCs did not induce proliferation (data not shown). We observed that LNSCs affected anti-CD3/28-induced T-cell proliferation in a ratio-dependent manner. In an LNSC/T-cell ratio of 1:2, a clear suppression of T-cell proliferation was observed, reflected by a lower number of divisions as measured by carboxyfluorescein succinimidyl ester dilution (Fig. 3a, *blue line*). Using the 1:2 LNSC/T-cell ratio, we found that LNSCs from healthy donors and individuals with RA risk induced a significantly higher number of CD4^+^ and CD8^+^ T cells that divided only once and then stopped proliferating (Fig. 3b, *red bars*), when compared with stimulated T cells in the absence of LNSCs. Of interest, this block in T-cell division was slightly diminished in co-cultures of T cells with RA LNSCs; however, variability between donors was high. In contrast, when T cells were cultured with LNSCs from healthy control subjects in ratios of 1:5 and 1:10 (Fig. 3a, *orange* and *pink lines*), we observed a stimulatory effect on T-cell proliferation, as observed by a significantly lower frequency of undivided T cells (Fig. 3b, *black bars*), when compared with stimulated T cells in the absence of LNSCs. Notably, this stimulatory effect when using LNSC/T-cell ratios of 1:5 and 1:10 was less clear in co-cultures with LNSCs from individuals with RA risk or patients with RA.

### NO production by LNSCs is similar between healthy subjects, individuals with RA risk and patients with RA

We also incubated LNSCs for 48 h with IFN-γ prior to co-culture with T cells. IFN-γ produced by activated T cells is known to play a key role in triggering NO production by LNSCs and therefore in increasing their immunosuppressive potential [20, 32, 33]. We found that pre-incubation with IFN-γ indeed slightly boosted the inhibitory capacity of LNSCs (as observed in the 1:2 LNSC/T-cell ratio) for most of the donors tested, including RA LNSCs (Fig. 3b). Measurement of NO in co-culture supernatants revealed a significantly higher NO production (*P* = 0.001) in the situation in which LNSCs and T cells were mixed in a 1:2 ratio when compared with the 1:40 ratio (Fig. 3c). However, this significant increase was observed similarly in all donor groups, and IFN-γ stimulation did not show an additional effect. This indicates that the increase in NO was probably due to a higher density of LNSCs and was not related to the altered T-cell proliferation observed when co-cultured with RA LNSCs.

## Discussion

In this study we set up an experimental model using human LNSCs to allow research on the role of the human LN microenvironment during health and RA. The in vitro expanded human LNSCs express key characteristics as described earlier in mice [16, 34]. During passaging these markers stay relatively stable, and their expression and induction are largely independent of podoplanin expression, the main marker for FRCs. The frequency of podoplanin-positive cells varied during culturing and between donors, but without any consistent trend towards loss of increase over passages. This variation was observed especially in those LNSC cultures containing high percentages of both FRC and DN cells, therefore probably reflecting a preferential outgrowth of one subset over the other. Mouse studies have shown that DN cells and FRCs resemble each other but have a differential expression of adhesion molecules and IL-7, which in mice are exclusively expressed in FRCs [16]. Our study showed similarities, because basal IL-7 mRNA levels correlated positively with basal podoplanin mRNA levels, and ICAM-1 induction appeared to be co-regulated with podoplanin induction as also observed in mice [34]. However, we also demonstrate that LNSC cultures consisting of cells with low podoplanin expression can also express relatively high levels of IL-7. In addition, in human LNSC cultures DN cells are relatively more abundant than FRCs as described before [24]. Together, these findings suggest a comparable role of DNs and FRCs in humans, although additional studies using isolated LNSC subsets are essential to prove this.

Interestingly, the expression of CXCL12, a B-cell chemoattractant [35], was significantly lower in LNSCs derived from individuals with RA risk than in healthy control subjects. CXCL12^+^ stromal cells derived from both bone marrow and tonsils (LN-like FRCs) of healthy donors can attract malignant B cells and appear to enhance the survival of follicular lymphoma B cells compared with healthy B cells isolated from blood [36]. Similarly, B-cell survival in the synovium is dependent on IL-6 and CXCL12, which are overexpressed by RA synovial fibroblasts [37]. The lower CXCL12 expression in LNSCs of individuals with RA risk might reflect an attempt to prevent autoreactive B cells from accessing the LN and impair their survival. We also detected a lower induction of CCL19 and CXCL13 after stimulation with TNF-α plus lymphotoxin α_1_β_2_ in LNSCs derived from patients with RA. Overall, these data suggest that LNSCs of individuals with RA-specific systemic autoimmunity display an altered chemokine profile, which may lead to disturbed trafficking of lymphocytes within the LN. Future studies are needed to confirm this and to investigate the mechanism by which chemokine production is disturbed in autoimmune LNSCs.

Next to lymphocyte trafficking and survival, LNSCs play a crucial role in regulating adaptive immune responses. LNSCs prevent extensive T-cell proliferation and thereby dampen immune responses through the release of NO in a tightly regulated and contact-dependent manner [20, 32, 33]. Our results diverge from murine studies where low numbers of LNSCs already lead to full inhibition and NO plays a crucial role [20, 32, 33]. We observed incomplete suppression when an LNSC/T-cell ratio of 1:2 was used and found that proliferation was even increased in LNSC/T-cell ratios of 1:5 and 1:10. Even though NO levels are higher in an LNSC/T cell ratio of 1:2, no differences were observed between donor groups. Therefore, the altered suppressive effect observed in RA LNSCs at the ratio of 1:2 is probably not dependent on changes in NO production. Likewise, the expression of IL-7, measured at P2 under homeostatic conditions that might drive T-cell proliferation, was not differentially expressed between donor groups or correlated with T-cell proliferation (data not shown) [38]. Even though IFN-γ signalling on LNSCs is crucial for NO production, exogenous IFN-γ alone increases only *NOS2*, the gene encoding inducible nitric oxidase synthase, but it fails to increase NO or nitrite in culture medium [32]. Furthermore, Transwell experiments show that LNSC-T-cell contact is needed to induce NO production and consequently T-cell suppression [20, 32, 33]. Taken together, this points towards additional components in this pathway derived from intimate cell contact to constrain T-cell proliferation, but future research is needed to formally prove this contact dependency in human co-cultures. Furthermore, in contrast to murine studies, which mostly use sorted and autologous cells derived from T-cell receptor (TCR) transgenic mice, we used an allogeneic co-culture system. Missing TCR-major histocompatibility complex (TCR-MHC) interaction might diminish close cell-cell contact, or mismatched TCR-MHC might additionally trigger T-cell proliferation [39]. However, our observations in healthy LNSCs are in line with data derived from mesenchymal stem cells (MSCs). In mice as well as in humans, using an allogeneic system containing MSCs and T cells, suppression of T-cell proliferation was observed only when relatively high numbers of MSCs were used, whereas relatively low numbers of MSCs supported T-cell proliferation [40, 41]. Furthermore, co-culture of allogeneic T cells with human MSCs in MSC/T-cell ratios of 1:4 and 1:40 increased the numbers of FoxP3-expressing cells [40], and maintenance of regulatory T cells by LNSCs has also been observed in mice [17]. It will be interesting to investigate in future experiments whether human LNSCs also play a role in maintenance of regulatory T cells and whether this process is disturbed in LNSCs from patients with RA. However, these experiments are highly challenging, because for human studies, knowledge is lacking on well-defined self-antigens expressed by human LNSCs and the availability of corresponding autoreactive human T cells.

The suppression of T cells in low LNSC/T-cell ratios (1:2) and the immunostimulatory effect in higher ratios (1:5 and 1:10) was seen mostly in LNSCs from healthy individuals. In this study, we show, for the first time to our knowledge, that this bipolar behaviour depending on LNSC/T-cell ratio is less maintained in LNSCs derived from patients with RA. It is tempting to speculate that reduced inhibition of T cells might result in less inhibition of self-reactive T cells and that reduced proliferation or induction of regulatory T cells leads to loss of tolerance. This and the cellular mechanism behind the potential exhausted state and aberrant function of RA LNSCs remain to be determined in future studies.

## Conclusions

Overall, we developed, for the first time to our knowledge, an experimental model to study the role of human LNSCs during the earliest phases of RA. Our exploratory study shows differences between the LN microenvironment of individuals with RA risk, patients with RA and healthy control subjects. To study in detail their immunoregulatory function, in vitro expansion of LNSCs is required. Because it is difficult to obtain LN biopsies from a large cohort of individuals with RA risk and patients with RA, and because the culture of human LNSCs is very time-consuming owing to their slow growth, the number of donors analysed in this study is relatively low. Also, because of the high inter-individual variation in podoplanin expression, the contribution of different LNSC subsets to the findings reported here remains to be further explored. The translation from in vitro results to in vivo relevance should be demonstrated by using mouse models or through targeted intervention studies in patients. However, using this in vitro model, we can start delineating the role of human LNSCs in T-cell-mediated B-cell responses during the earliest phases of RA, which ultimately may lead to the identification of innovative targets for immunomodulation.

## Additional file

Additional file 1: **Figure S1.** Gene expression levels over passages. The expression levels of VCAM-1, ICAM-1, IL-7 and PDPN (podoplanin) in LNSCs obtained from different passages was assessed by qPCR. Relative quantity (RQ) of 15 donors (*n* = 5 per donor group) is displayed. **Figure S2.** Correlation between podoplanin and IL-7 mRNA at P2. The expression levels of PDPN (podoplanin) and IL-7 were assessed by qPCR in passage 2 LNSCs (*n* = 61; donor characteristics in Table 1) and showed a positive correlation, which was not observed for other genes measured in these cells. Relative quantity (RQ) values were analysed by Spearman’s correlation test. **** *P* < 0.0001. **Figure S3.** Induction of genes characteristic for LNSCs. The expression levels of VCAM-1, ICAM-1, IL-7 and PDPN (podoplanin) was assessed by qPCR in LNSCs (passages 4 to 8) after stimulation with TNF-α and lymphotoxin α_1_β_2_ for 4 h and 24 h. Mean fold induction (FI) and SD of *n* = 5 per donor group are shown (donor characteristics in Table 2). The dotted line represents a fold induction of 1. **Figure S4.** Correlation between podoplanin and ICAM-1 induction. The upregulated mRNA levels of PDPN and ICAM-1 upon stimulation with TNF-α and lymphotoxin α_1_β_2_ showed a strong positive correlation, which was not observed for VCAM-1 and IL-7. Fold induction values were analysed by Spearman’s correlation test. **** *P* < 0.0001. **Table S1.** Primers used in this study. (DOCX 248 kb)

## Abbreviations

ACPAs: Anti-citrullinated protein antibodies
ANOVA: Analysis of variance
APC: Allophycocyanin
BAFF: B-cell-activating factor
CCL: C-C motif chemokine ligand
cDNA: Complementary DNA
CFDA-SE: Carboxyfluorescein diacetate succinimidyl ester
CRP: C-reactive protein
CSFE: Carboxyfluorescein succinimidyl ester
CXCL: C-X-C motif chemokine ligand
DAS28: Disease Activity Score in 28 joints
DMARD: Disease-modifying anti-rheumatic drug
DN: Double-negative
ESR: Erythrocyte sedimentation rate
FACS: Fluorescence-activated cell sorting
FI: Fold induction
FITC: Fluorescein isothiocyanate
FRC: Fibroblastic reticular cell
FSC: Forward scatter
HEPES: 4-(2-hydroxyethyl)-1-piperazineethanesulfonic acid
ICAM-1: Intercellular adhesion molecule 1
IFN-γ: Interferon-γ
Ig: Immunoglobulin
*IgM-RF*: Immunoglobulin M-rheumatoid factor
IL: Interleukin
LN: Lymph node
LNSC: Lymph node stromal cell
LTβR: Lymphotoxin-β α_1_β_2_ receptor
MHC: Major histocompatibility complex
mRNA: Messenger RNA
MSC: Mesenchymal stem cell
NO: Nitric oxide
NSAID: Non-steroidal anti-inflammatory drug
PBA: Protein blocking agent
PBMC: Peripheral blood mononuclear cell
PE-Cy7: Phycoerythrin-cyanine 7
RA: Rheumatoid arthritis
RF: Rheumatoid factor
RQ: Relative quantity
SJC: Swollen joint count
SSC: Side scatter
TCR: T-cell receptor
TJC: Tender joint count
TNF-α: Tumour necrosis factor-α
ULN: Upper limit of normal
VCAM-1: Vascular cell adhesion molecule 1
