# Supplementary material for: Impaired lymph node stromal cell function during the earliest phases of rheumatoid arthritis
Source: Arthritis Res Ther. 2018 Feb 26;20:35. doi: 10.1186/s13075-018-1529-8 (PMC5828373; doi:10.1186/s13075-018-1529-8)
Supplement: Supplementary file 1 — Figure S1. Gene expression levels over passages. The expression levels of VCAM-1, ICAM-1, IL-7 and PDPN (podoplanin) in LNSCs obtained from different passages was assessed by qPCR. Relative quantity (RQ) of 15 donors (n = 5 per donor group) is displayed. Figure S2. Correlation between podoplanin and IL-7 mRNA at P2. The expression levels of PDPN (podoplanin) and IL-7 were assessed by qPCR in passage 2 LNSCs (n = 61; donor characteristics in Table 1) and showed a positive correlation, which was not observed for other genes measured in these cells. Relative quantity (RQ) values were analysed by Spearman’s correlation test. **** P < 0.0001. Figure S3. Induction of genes characteristic for LNSCs. The expression levels of VCAM-1, ICAM-1, IL-7 and PDPN (podoplanin) was assessed by qPCR in LNSCs (passages 4 to 8) after stimulation with TNF-α and lymphotoxin α1β2 for 4 h and 24 h. Mean fold induction (FI) and SD of n = 5 per donor group are shown (donor characteristics in Table 2). The dotted line represents a fold induction of 1. Figure S4. Correlation between podoplanin and ICAM-1 induction. The upregulated mRNA levels of PDPN and ICAM-1 upon stimulation with TNF-α and lymphotoxin α1β2 showed a strong positive correlation, which was not observed for VCAM-1 and IL-7. Fold induction values were analysed by Spearman’s correlation test. **** P < 0.0001. Table S1. Primers used in this study. (DOCX 248 kb) [file 13075_2018_1529_MOESM1_ESM.docx]

**Additional file 1**

**Impaired lymph node stromal cell function during the earliest phases of rheumatoid arthritis**Janine S Hähnlein, Reza Nadafi, Tineke de Jong, Tamara H Ramwadhdoebe , Johanna F Semmelink, Karen I Maijer, IJsbrand A Zijlstra, Mario Maas, Danielle M Gerlag, Teunis B H Geijtenbeek, Paul P Tak, Reina Mebius, Lisa G M van Baarsen

**Supplementary figures and figure legends**

**

**

**Figure S1: Gene expression levels over passages**The expression level of VCAM-1, ICAM-1, IL-7 and PDPN (Podoplanin) in LNSCs obtained from different passages was assessed by qPCR. Relative quantity (RQ) of 15 donors (n=5 per donor group) is displayed.

**
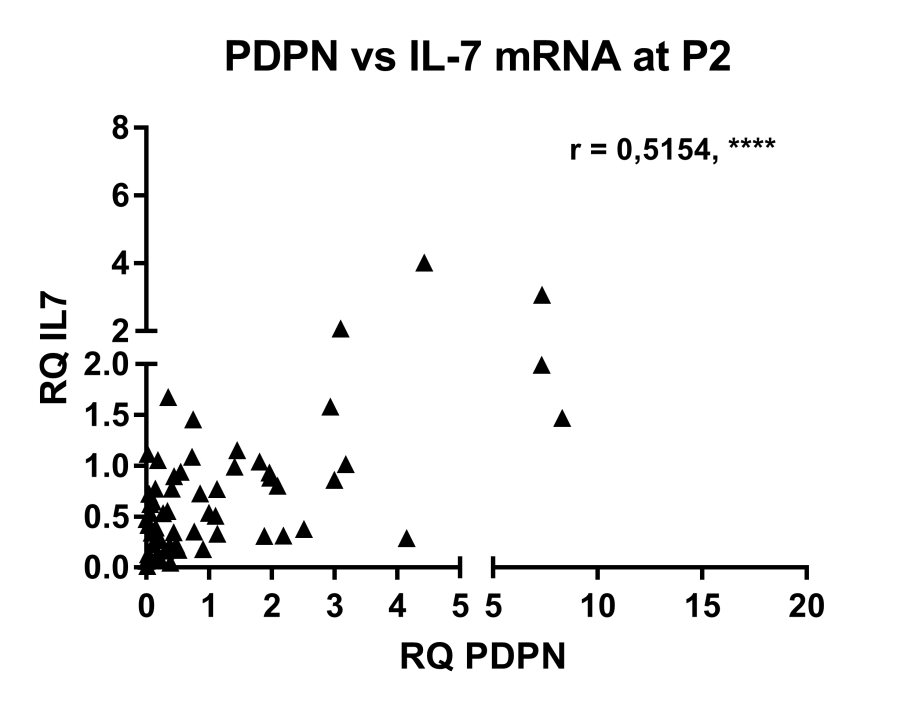
**

**Figure S2: Correlation between Podoplanin and IL-7 mRNA at P2**The expression levels of PDPN (Podoplanin) and IL-7 was assessed by qPCR in passage 2 LNSCs (n=61, donor characteristics in Table 1) and showed a positive correlation, which was not observed for other genes measured in these cells. Relative quantity (RQ) values were analysed by Spearman correlation test. **** p<0.0001

**

**

**Figure S3: Induction of genes characteristic for LNSCs**

The expression level of VCAM-1, ICAM-1, IL-7 and PDPN (Podoplanin) was assessed by qPCR in LNSCs (passages 4 to 8) after stimulation with TNFα and lymphotoxin α_1_β_2_ for 4h and, 24h. Mean fold induction (FI) and standard deviation of n=5 per donor group is shown (donor characteristics in Table 2). The dotted line represents a fold induction of 1.

**

**

**Figure S4: Correlation between Podoplanin and ICAM-1 induction**The upregulated mRNA levels of PDPN and ICAM-1 upon stimulation with TNFα and lymphotoxin α_1_β_2_ showed a strong positive correlation, which was not observed for VCAM-1 and IL-7. Fold induction values were analysed by Spearman correlation test. **** p<0.0001.

**Table S1:** Primers used in this study.

| **Gene symbol** | **Taqman ID** |  |
| --- | --- | --- |
| IL-7 | Hs00174202_m1 |  |
| LTβR | Hs00158922_m1 |  |
| ICAM-1 | Hs00164932_m1 |  |
| CXCL12 (SDF1) | Hs00171022_m1 |  |
| CCL19 | Hs00171149_m1 |  |
| CCL21 | Hs00989654_g1 |  |
| CXCL13 | Hs00757930_m1 |  |
| 18S RNA | Hs99999901_s1 |  |
| **Gene symbol** | **Forward primer Sequence 5’-3’** | **Reverse Primer Sequence 5’-3’** |
| VCAM-1 | CCGAAAGGCCCAGTTGAAG | AGCACGAGAAGCTCAGGAGAA |
| IL-6 | GACAGCCACTCACCTCTTCA | CCTCTTTGCTGCTTTCACAC |
| 18S RNA | CCGAGTAAGTGCGGGTCATAA | CCATCCAATCGGTAGTAGCG |
